# Effects of muscle relaxants on ischaemia damage in skeletal muscle

**DOI:** 10.1038/s41598-018-24127-2

**Published:** 2018-04-11

**Authors:** Thomas Ledowski, Simone Nißler, Manuel Wenk, Esther M. Pogatzki-Zahn, Daniel Segelcke

**Affiliations:** 1 Anaesthesiology Unit, Medical School, The University of Western Australia, 35 Stirling Highway, Crawley, WA 6009 UK; 2 Department for Anaesthesiology, operative Intensive Care and Pain Medicine, University Hospital Muenster, Albert-Schweitzer-Campus 1, A1, 48149 Muenster, Germany

## Abstract

Muscle ischaemia is frequently induced intraoperatively by i.e. a surgical tourniquet or during the re-grafting phase of a free muscle transplant. The resulting muscle cell damage may impact on postoperative recovery. Neuromuscular paralysis may mitigate the effects of ischaemia. After ethics approval, 25 male Sprague-Dawley rats were anaesthetized and randomly assigned to 1 of 4 groups: Sham operation, treatment with normal saline, treatment with rocuronium (muscle relaxant) 0.6 or 1 mg kg^−1^, respectively. In the non-sham groups, ischaemia of one hind leg was achieved by ligation of the femoral vessels. Muscle biopsies were taken at 30 and 90 min, respectively. Cell damage was assessed in the biopsies via the expression of dystrophin, free calcium, as well as the assessment of cell viability. Pre-ischaemia muscle relaxation led to a reduction in ischaemia-induced muscle cell damage when measured by the expression of dystrophin, cell viability and the expression of free calcium even after 90 min of ischaemia (i.e. ratio control/ischaemic site for dystrophin expression after saline 0.58 ± 0.12 vs. after 1 mg/kg rocuronium 1.08 ± 0.29; P < 0.05). Muscle relaxation decreased the degree of ischaemia-induced muscle cell damage. The results may have significant clinical implications.

## Introduction

Skeletal muscle ischaemia is frequently induced during surgery either by means of a tourniquet or during microvascular free flap procedures. Focal and regional muscle fibre necrosis and degeneration have been observed distally as well as underneath of applied surgical tourniquets^1^, and the ischemia-induced muscular damage may impair patient recovery^2^. For example, patients in whom a tourniquet had been used during total knee arthroplasty showed a diminished quadriceps muscular strength for up to 3 months^3^. In this context is the degree of ischaemia-induced muscle damage strongly associated with the duration of ischaemia^4^.

Though for this reason, surgeons will always attempt to keep ischaemia times as short as possible, not infrequently this may not be achievable due to unexpectedly difficult surgical conditions. It is thus desirable to identify additional strategies to decrease the degree of muscular damage during prolonged periods of intra-operative ischaemia.

Neuromuscular blocking agents (NMBA), such as rocuronium, are worldwide used as a component of general anaesthesia. These drugs competitively block the nicotinic acetylcholine receptor on the muscle cell membrane^5^. As a flow-on effect from the resulting muscle paralysis, oxygen consumption is reduced within the affected muscle, an effect which can be measured in clinical scenarios^6^. On a cellular level, a paralysis-provoked reduced metabolic activity may provide a degree of protection from the effects of ischaemia by reducing muscle cell damage; however, such an indirect protective effect of NMBA on muscle outcome after surgery has never been investigated. In this context, we hypothesized that muscle relaxation prior to the induction of intraoperative ischaemia may mitigate the cell damage in affected muscles.

Thus, the aim of this study was to investigate, whether the use of the NMBA rocuronium prior to experimentally-induced ischaemia could be a potential pathway to mitigate the effects of ischaemia on skeletal muscle.

## Material and Methods

### General

The experiments in this study were reviewed and approved by the institution’s Animal Ethics Committee of the State Agency for Nature, Environment and Consumer Protection North Rhine-Westphalia (LANUV), Recklinghausen, Germany and all experiments were performed in accordance with this approval. Male Sprague-Dawley (SD) rats (Charles River, Germany), aged 6 to 8 weeks (204 g ± 21 g), were used; the animals were housed in ventilated plastic cages (GR1800 Double Decker for Rats, Techniplast, Italy) with environmental enrichment under a 12/12 h light/dark cycle, in groups of 3 animals, with *ad libitum* access to food and water. At the end of the experiments, all animals were euthanized by decaptitaion under isoflurane anaesthesia. Rats (total n = 25) were randomly assigned to sham (control-group = animals instrumented, but no drug, no ischaemia; n = 6), saline-treated (vehicle-group; n = 6), moderate-dose Rocuronium (0.6 mg kg^−1^; n = 5) or high-dose Rocuronium (1 mg kg^−1^; n = 8) groups. The sample size analysis (target group size 8 animals) was performed a priori with the software G-Power 3.1.9.2 (http://www.gpower.hhu.de/). The different group size described in the results section was caused by the premature death of some randomized animals. All experiments commenced at 8 a.m. and all persons performing the experiments detailed below were blinded as to the drug/dose/saline allocation of the involved animals.

### Model for muscle ischaemia

All rats received inhalational induction of anaesthesia using isoflurane. For anaesthesia, a mixture of isoflurane (5% for induction and 1.5–2.0% for maintenance) in O_2_:N_2_O (30:70) was used. Buprenorphine (0.05 mg kg^−1^) was subsequently injected subcutaneously with an incubation time of 30 minutes to achieve adequate analgesia during the experiment, because Isofluran does not have a sufficient analgesic effect. The doses and incubation times for buprenorphine were in accordance to previously published experimental designs^7,8^. To avoid a drop in the body temperature, the animals were placed on a water-perfused heating mat. A continuous temperature measurement was achieved via a rectal temperature probe, and a core temperature of 35.5–37 °C was maintained. Oxygen saturation and heart rate were monitored via pulse oximetry on the left front paw. Continuous capnography was utilized, and the endtidal CO_2_ kept between 20–35 mmHg.

After induction of anaesthesia, an initially performed tracheostomy was followed by mechanical ventilation (respiration rate 60, tidal volume 8–10 ml kg^−1^), to ensure controlled respiration under clinical conditions. In order to later assess the degree of muscle relaxation (see details below), the gracilis muscle of both hind legs was visualized via a skin incision. Subsequently, the femoral vein and femoral artery of both hind legs were dissected, and a loop of silk 4–0 (surgical suture material) placed loosely around each femoral plexus (all groups).

**Figure 1:**
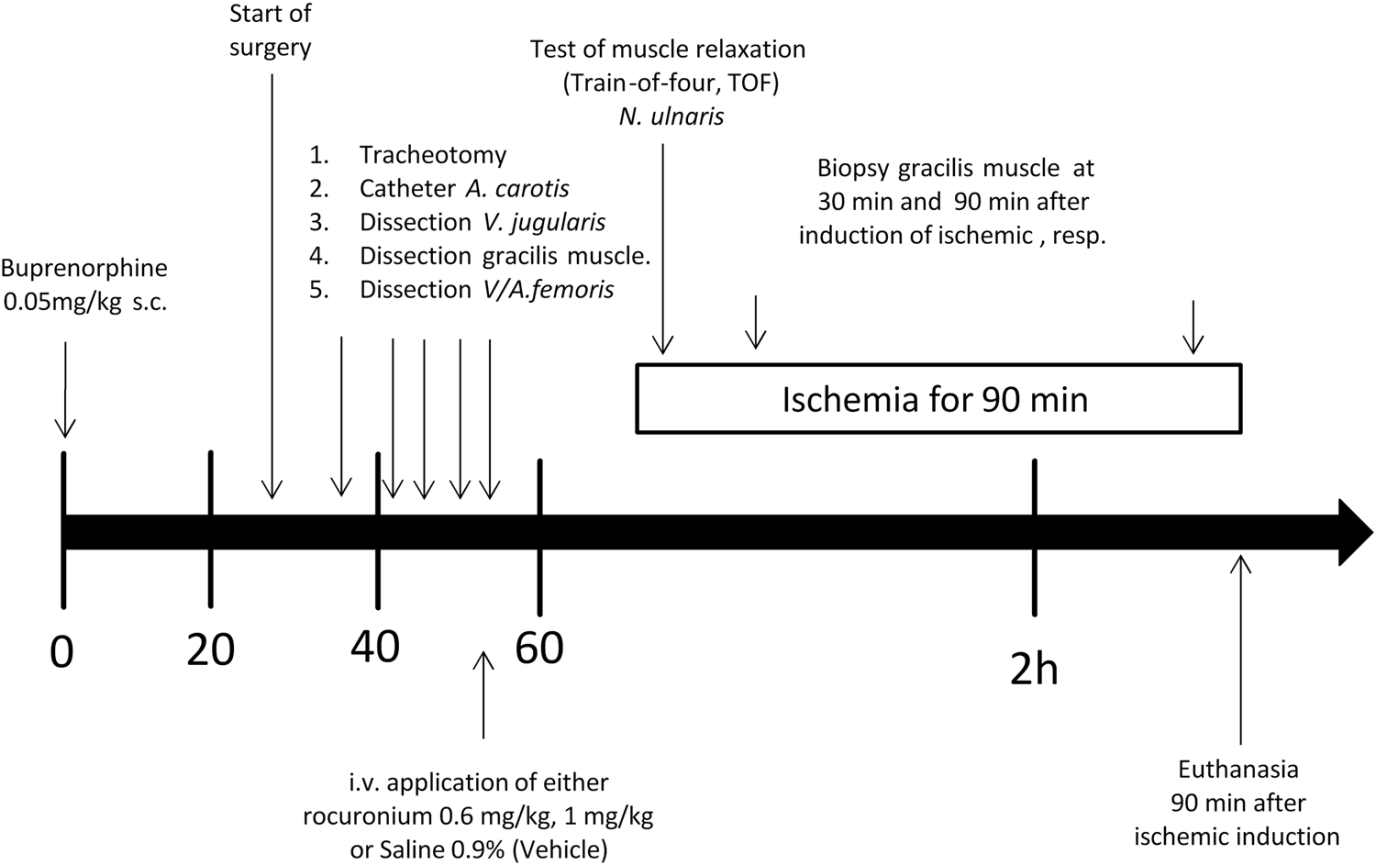
Flowchart of experimental design. Male Spargue Dawley (SD) rats were subjected to 90 min of ischaemia and finally euthanized. During the 90 min of ischaemia, rats were kept under general anaesthesia with isoflurane and buprenorphine. To achieve ischaemia, both femoral artery and vein were ligated with 4-0 silk. In addition, the right internal jugular vein was cannulated and used for the administration of the investigated drugs. For further details see the material and methods section.

Figure 1 provides a time line of the experiments.

### Muscle relaxation and assessment of neuromuscular block

Thereafter, the baseline degree of muscle relaxation before the application of any substances (Rocuronium or Vehicle [saline 0.9%]) was measured at the gracilis muscle via “train-of-four” (TOF) test (2 Hz ulnar nerve stimulation with 2 mA current). The assessment of the TOF describes a series of 4 electrical nerve stimuli and the observation of the resulting muscle twitches. In NMBA, if all 4 twitches are of equal strength, a state of non-paralysis may be assumed. If no twitch is observed, muscle relaxation is deep, and the return of the first to fourth twitch (1/4–4/4) reflects the recovery from the effects of a NMBA. A weakening observed from the first to the fourth twitch during this process reflects the ongoing blockade of nicotinic acetylcholine receptors at the presynaptic nerve terminal – this results in a lack of positive feedback to the stimulated nerve which in turn does not produce sufficient acetylcholine to provoke 4 muscle twitches of equal strength. This so-called *fade* reflects the later stage of recovery from NMBA effects. The use of TOF stimulation in rodents is well described^9^. Once the state of “non-paralysis” had been confirmed, muscle relaxant (one of two doses: rocuronium 0.6 and 1 mg kg^−1^, ESMERON^®^, Essex Pharma, Germany) or saline 0.9% were injected intravenously (jugular vein) according to the group allocation (see above). Rocuronium is an amino-steroidal muscle relaxant achieving (as all other NMBA in clinical use) its effect by means of a competitive antagonism at the nicotinic acetylcholine receptor at the post-synaptic muscle cell membrane (main effect) as well as the pre-synaptic nerve terminal (minor effect). Rocuronium was introduced worldwide in the mid 1990s and is one of the most widely used NMBA in human as well as animal anaesthesia. *In vivo*, rocuronium must be administered intravenously or intramuscularly and, similar to curare, has no effect when taken orally or when topically applied. Rocuronium is not known to show a significant interaction with the analgesic buprenorphine used in this experiment. The doses used in this experiment were in-line with doses used in humans but also in rat-based studies^10^.

90 seconds after the drug administration, the electric stimulation of the ulnar nerve was repeated as above in order to confirm a sufficient neuromuscular block (TOF count of max. 2/4 twitches; equalling a frequently targeted depth of neuromuscular blockade during surgery in humans). Once sufficient muscle relaxation (or no relaxation in the sham and saline groups) had been confirmed by a person not performing the analysis of the muscle biopsies, the femoral plexus on one leg (=ipsilateral) were ligated using a 2-0 silk loop. This time point defined the onset of ischaemia. The contralateral side served as the non-ischaemic control side. For further analysis, 0.6 mm punch biopsies from the gracilis muscles of both the ipsilateral as well as contralateral (non-ischemic) leg were taken at 30 and 90 min post-ischaemia induction of the ipsilateral leg. These biopsies were processed for histological- (paraffin and cryo-embedding) and molecular biological analysis (Western blot, MTT assay; details see below). After the last biopsy, all animals were euthanized by decapitation under isoflurane anaesthesia.

### Assessment of muscle cell integrity (Dystrophin assay)

Dystrophin is one of the integral membrane proteins in the skeletal and cardiac myocytes^11^. Experimental ischaemia conditions lead to a specific loss of dystrophin in the myocyte membrane^12^. The expression of Dystrophin in the gracilis muscle was determined by Western blot analysis at different time points (30 and 90 min) after induction of ischaemia and the results were compared with sham-treated (fully instrumented, but no drugs and no ischaemia-induced on either leg) animals as a control. After homogenization in a buffer containing a cocktail of proteinase inhibitors, proteins (protein concentration was 60 µg, quantified by the Bradford method) were separated by 10% SDS-polyacrylamide gel electrophoresis (Bio-Rad Laboratories, Munich, Germany) and transferred to nitrocellulose membranes (Amersham-Bioscience, Freiburg, Germany) by semi-dry blotting. The membranes were blocked with 8% milk powder in TBS. The antibody against Dystrophin (15277, Abcam, UK) was used at a dilution of 1: 1000. The protein β-actin (1:50.000. A2066, Sigma, Germany) was used as loading control. The calculations were carried out using a digital system from Peqlab. The densiometric ratios of the signals of Dystrophin were generated with the sham group.

For the immunohistochemical staining of dystrophin, the tissue samples were removed and post-fixed for 24 hours in 4% PFA, followed by a 3 hour wash with 0.1 M phosphate-buffered saline (PBS) and cryopreserved in 0.4 M sucrose and 0.8 M sucrose overnight at 4 °C. The tissues were embedded in Tissue-Tek^®^O.C.T.™ (Sakura Finetek, Germany), compound and stored for at least 3 days at −80 °C.

The slices (10 µm) were incubated in acetone for 10 min and afterward dried for 30 min in the air. Thereafter, the tissue samples bordered with a PAP pen (2601, Abcam, UK), washed in 0.1 M PBS 3 times for 10 min and subsequently blocked 1 h at room temperature with 5% normal goat serum in 0.1 M PBS and 0.3% Triton x-100. The polyclonal Dystrophin antibody (15277, Abcam, UK) was diluted 1:200 in blocking solution and incubated for 16–24 h at 4 °C. Thereafter, the slices were washed three times with PBS for 10 min and the second antibody, goat anti-rabbit Alexa Fluor 488 (1:1000 in PBS, AF488, 150077, Abcam, UK) for 1 h at room temperature, was applied. After three washes for 10 min, the slices were dried for 15 min and after covered up with confocal-matrix® (Micro-Tech-Lab, Germany). The images were performed by Zeiss Apotom 2 and the Zen software (Zeiss, Germany).

### Assessment of free Calcium (Alizarin red S staining)

For the visualization of free Calcium under ischemia conditions an alizarin red s staining^13^ was used. An accumulation of intracellular free Calcium is a marker for ischaemia injury of skeletal muscle^14^. The paraffin embedded muscle preparations were cut into 5 µm thick slices. The slices were de-paraffining with xylene 2 times for 2 min. Afterward, the slices were cleared and hydrating with descending alcohol concentration from 100%, 95% to 50% and final with distilled water for 2 min. For Alizarin red s (0348.1, Roth, Germany) staining was used a stock solution (2 g.100 ml^−1^, pH 4.1–4.3) with an incubation time of 10 min. The slices were dehydrated with acetone for 20 sec and acetone-xylene solution (50:50) for another 20 sec. Thereafter, the slices were incubated with xylene for 5 min and covered with Entellan. The muscle preparations were photographed 3 times on random sites with a digital Zeiss microscope and with a magnification of 20x. The staining of free calcium was determinate with threshold colour by the open source software *ImageJ* (https://imagej.nih.gov/ij/). The hue control for all pictures was defined from 0 to 19, the interval for saturation and brightness were not limited (0–255). The labelled particles were evaluated by the analysing tool in ImageJ with following adjustments, Size (pixel^2^) was from 2-Infinitiy. The area of the labelled particles was converted into the percentage and the ratio generated to control group (sham).

### Assessment of muscle fibre vitality (MTT-Assay)

For measurement of cell vitality of the muscle samples, we utilized a MTT-(3-(4,5-Dimethyl-2-thiazolyl)-2,5-diphenyl-tetrazoliumbromid) assay, which was derived from cell culture methods. The muscle samples were homogenized and diluted (1:10) with 0.2 M sucrose solution. From this solution, 1 ml was incubated with 200 m MTT 0.5% for 90 min at 37 °C. The pigment extraction was achieved by mixture with 300 U × min^−1^ at 37 °C in 1 ml isopropanol for 60 min. The probes were centrifuged with 5000 U × min^−1^ for 10 min at 4 °C. The extinction supernatant was evaluated at a wavelength of 490 nm.

### Statistics

Results of the Dystrophin expression, cell metabolic activity, and measurement of free calcium were compared by nonparametric analysis, including the Friedman test for within-group comparisons and the Kruskal-Wallis test for between-group comparisons. Multiple comparisons after the Friedman test and Kruskal-Wallis test were performed by a Dunnett´s test for nonparametric analysis, respectively. A P < 0.05 was considered significant. Data were analyzed by Prism software, version 5.02 (GraphPad, San Diego, USA). All data comparing the expression of dystrophin, cell viability (MTT assay) and the expression of free calcium after pre-treatment with saline vs. rocuronium 0.6 or 0.9 mg kg^−1^, respectively, are displayed as the ratio of ischaemic/control (non-ischaemic) site from the same animal.

### Data availability

The datasets generated during and/or analysed during the current study are available from the corresponding author on reasonable request.

## Results

### Muscle relaxation with rocuronium prevents ischaemia-induced down-regulation of dystrophin

**Figure 2:**
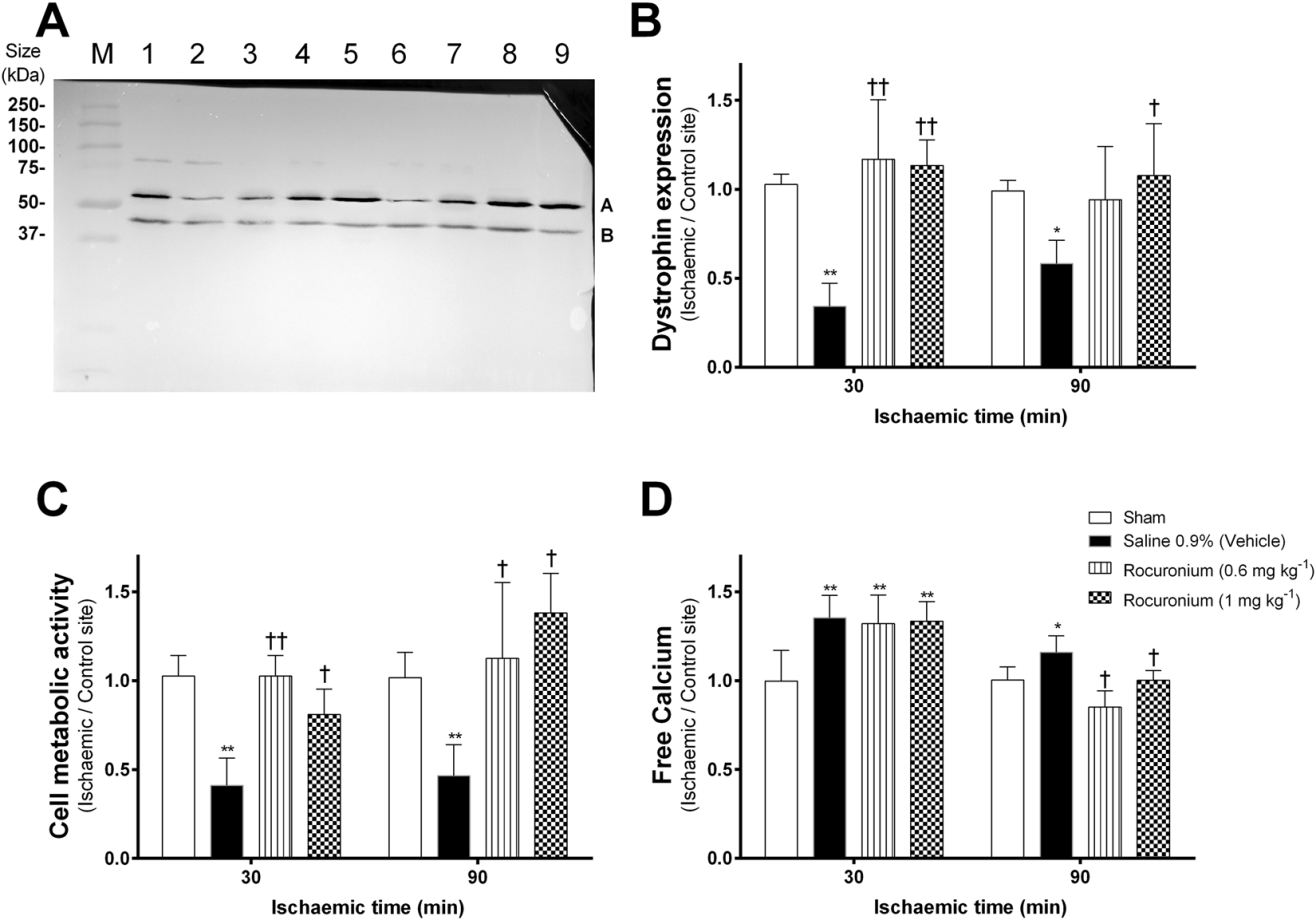
Effect of the intravenous administration of rocuronium or saline on the expression of dystrophin-protein (**B**) (Western blot, representative Blot (**A**), the cell metabolic activity (MTT-assay) (**C**), and the levels of cytosolic free calcium (Alizarin staining) (**D**) in ischaemic vs. non-ischaemic (control) gracilis muscle at 2 different time points (30 and 90 min post induction of ischaemia). Sham animals: instrumented but no ischaemia induced. mean ± SD; *P < 0.05, **P < 0.01 vs Sham; ^†^P < 0.05, ^††^P < 0.01 vs. Vehicle (saline). (**A**) M = marker, 1 = sham, 2 = Saline 0.9%, 3 = rocuronium 0.6 mg kg^−1^, 4 = rocuronium 1 mg kg^−1^, 5 = sham, 6 = Saline 0.9%, 7 = rocuronium 0.6 mg kg^−1^, 8 = rocuronium 1 mg kg^−1^, [1–4] 30 min ischaemic time, [5–8] 90 min ischaemic time.

**Figure 3:**
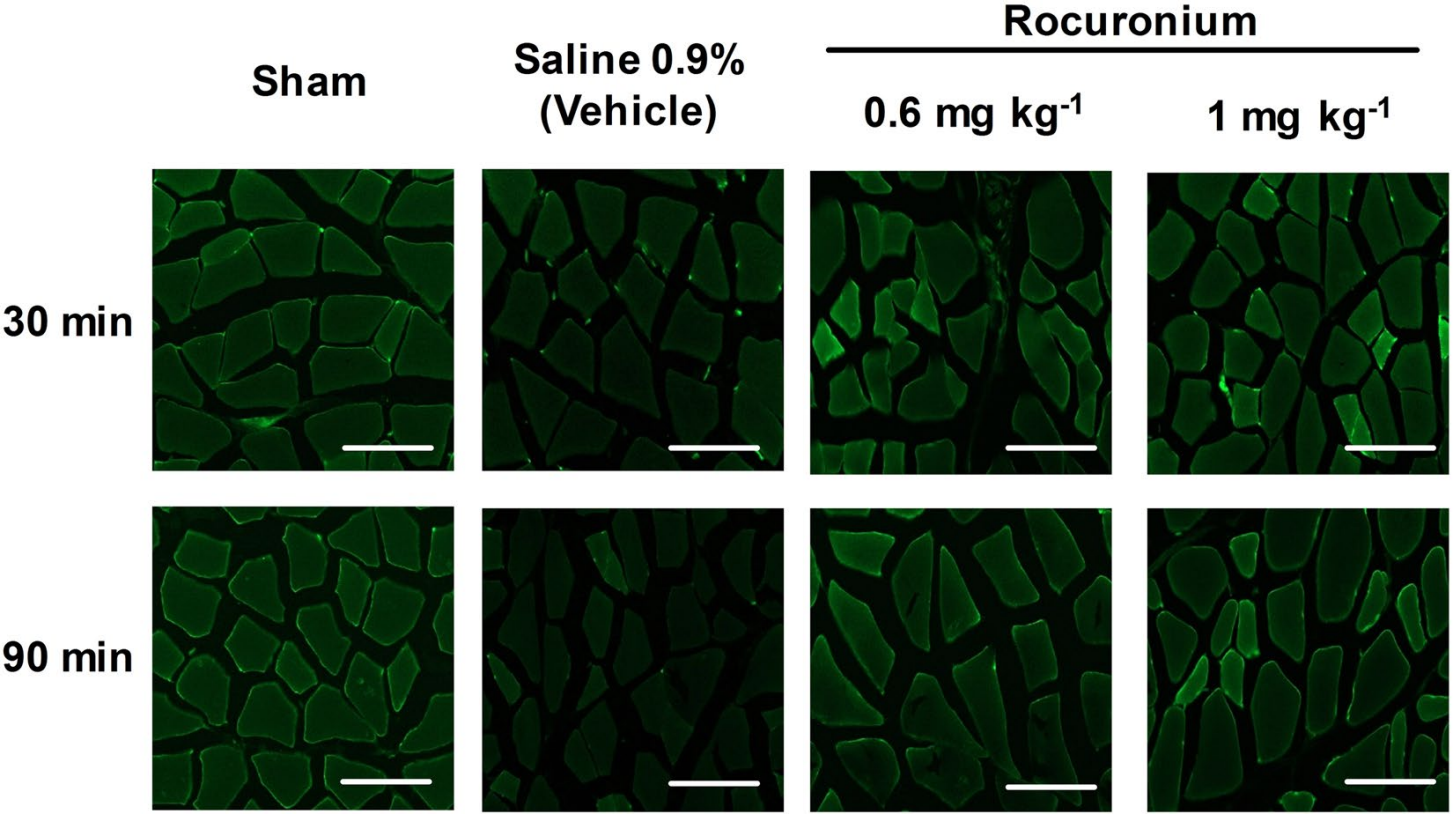
Exemplary immunoreactivity of Dystrophin (Dys) in the gracilis muscle 30 and 90 min after induction of ischaemia in animals receiving saline 0.9% (vehicle) vs. rocuronium 0.6 mg kg^−1^ or 1 mg kg^−1^, respectively. Weaker green fluorescence indicates increased disintegration of the cell membrane. Scale bar represents 50 µm.

In saline-treated rats, the ratio (ischemic/control site) of Dystrophin expression was significantly decreased to 0.34 ± 0.13 (Mean ± SD; P < 0.01) at 30 min and to 0.58 ± 0.12 at 90 min (P < 0.05) after induction of ischemia (Figs 2A and 3). In contrast, the application of rocuronium prevented the decrease of dystrophin expression or even increased this parameter (vs. saline): rocuronium 0.6 mg kg^−1^ (1.19 ± 0.36 at 30 min, P < 0.01; 0.94 ± 0.3 at 90 min) and rocuronium 1 mg kg^−1^ (1.13 ± 0.14 at 30 min, P < 0.01; 1.08 ± 0.29 at 90 min, P < 0.05).

### Pre-ischaemia muscle relaxation leads to increased cell metabolic activity

After induction of ischaemia, the muscle cell metabolic activity was significantly decreased (Fig. 2B) in the saline-treated animals at 30 min (0.41 ± 0.15, P < 0.01) and at 90 min (0.47 ± 0.18, P < 0.01) when compared to sham treated animals (1.03 ± 0.12 at 30 min; 1.02 ± 0.14 at 90 min). Muscle relaxation with 0.6 mg kg^−1^ and 1 mg kg^−1^ rocuronium (vs saline) significantly preserved the cell metabolic activity (0.6 mg kg^−1^: 1.03 ± 0.12 at 30 min, P < 0.01; 1.13 ± 0.43 at 90 min, P < 0.05; 1 mg kg^−1^: 0.81 ± 0.14 at 30 min, P < 0.05; 1.38 ± 0.22 at 90 min, P < 0.05).

### Rocuronium pre-treatment decreases ischaemic-induced free cytosolic calcium

The cytosolic free level of calcium was significantly increased (Fig. 2C) from 0.1 ± 0.17 in sham animals to 1.35 ± 0.16 (P < 0.01) in saline-treated rats at 30 min ischaemia, and from 1 ± 0.074 to 1.16 ± 0.09 at 90 min ischemia (P < 0.01), respectively. The intravenous pre-treatment with both different doses of Rocuronium significantly prevented the increase of free calcium at 90 min post-induction of ischaemia (0.6 mg kg^−1^: 0.85 ± 0.09, P < 0.05; 1 mg kg^−1^: 1 ± 0.06, P < 0.05), but not after 30 min.

Overall, different doses of rocuronium as well as the different time points of the muscle biopsies used in the study did not result in significantly different effects on the investigated muscle cells.

## Discussion

The present study indicates that neuromuscular paralysis with the NMBA rocuronium may have mitigating effects on the ischaemia-induced damage to skeletal muscle in rats. Rocuronium reduced ischaemia-induced down-regulation of dystrophin, the ischaemia-induced reduction in cell metabolic activity and prevented the increase of free cytosolic calcium.

Our experimental data suggest a protective role of an intraoperative neuromuscular block during surgery producing temporary ischaemia (i.e. tourniquet or vessel clamping during fee flap surgery). As NMBA are frequently utilized during general anaesthesia, the implementation of a (deeper) neuromuscular block prior to a surgically-induced ischaemia appears relatively easy to achieve. In fact, our data suggest a dose dependent effect supporting the provision of a deep neuromuscular block. The model of this study can neither prove this effect in humans, nor its clinical relevance. However, our data may nonetheless be useful as a translational approach to stimulate further related research, i.e. in a clinical setting.

The current study, to the best of our knowledge, is the first to describe an ischaemia-protective effect of NMBA on skeletal muscle on a cellular level. However, the underlying hypothesis of a clinically useful effect of NMBA on muscular oxygen consumption is nothing new. Vernon *et al*.^6^ found both oxygen consumption as well as energy expenditure significantly reduced after the use of NMBA in critically ill, mechanically ventilated children. However, the effect described by the authors was relatively small, and its clinical implication remains unclear. Possibly due to the relatively small effect or the low number of included subjects (n = 9), Lemson *et al*.^15^ were unable to replicate Vernon’s^6^ earlier findings in children after complex cardiac surgery. However, the included children were very young, therefore possibly having a low total muscle mass which may have further impaired detection of differences in global oxygen consumption. A further study investigating 17 children after cardiac surgery did describe a significant NMBA-provoked decrease of oxygen consumption in only a minority of subjects^16^. On a more localized level, Rhee *et al*.^17^ could not demonstrate an effect of muscle relaxation on ischaemia-induced muscular oxygen desaturation in a human isolated-forearm model. However, the authors discuss that the method of measurement (oxygen desaturation of forearm muscles via near infrared spectrometry) used in their study was relatively crude and therefore unlikely suited to detect subtle differences.

Based on these findings, it can be assumed that the NMBA effect on global oxygen consumption is relatively low and, at least in non-critically ill patients, possibly clinically insignificant. However, the effects on cell metabolic viability and retention of cell integrity may be significantly higher. Our results showed that cellular muscle integrity (as measured via the Dystrophin assay) was significantly compromised even after a relatively short period of ischaemia (30 min) when saline had been injected. However, pre-treatment (muscle relaxation) with rocuronium before induction of ischaemia resulted in a dose-dependent preservation of cell structural integrity. These effects were also reflected by the results of the MTT assay^18^ which indicated that the number of viable muscle tissue had not significantly been changed by ischaemia after pre-treatment with rocuronium, whereas pre-treatment with saline lead to a dramatic decrease of viable muscle tissue. In the saline group, we recorded an increase in the level of free cytosolic calcium at both 30 and 90 minutes post ischaemia induction. This observation may be explained by the ischaemia-provoked disruption of ATP-dependent calcium transport mechanisms^19^. Free cytosolic calcium, by means of over-stimulation of calcium-dependent signal transduction pathways, may further contribute to the destruction of cellular integrity^20^. The fact that we found the levels of free calcium decreased at 90 min after an initially recorded increase at 30 min may be explained by the reduction of this “vicious cycle” by means of rocuronium pre-treatment. This may have permitted the investigated muscle fibres to restore the calcium equilibrium at 90 min after an initial “shock” noted at 30 min. However, this assumption cannot be firmly proven by our study design. There were no differences between the different time points for both Dystrophin and MTT assays suggesting that the ischaemia-induced cell damage did not significantly progress over this period of time. However, these findings may not be indicative of any reperfusion injury (not investigated by us) after either 30 or 90 min ischaemia.

The results suggest a role for NMBA in the protection of muscle tissue from ischaemia-induced damage. Though not investigated by us, this may also have consequences for the degree of post-ischaemic reperfusion injury.

In a clinical scenario, and considering earlier findings of a tourniquet-associated reduced muscular strength for up to 3 months after surgery^3^, the intraoperative use of NMBA may have resulted in a better functional outcome. In the setting of the transplantation of a free muscle graft, duration of warm ischaemia is strongly correlated with the degree of muscle damage and the likelihood of graft-associated complications^21^. Deep paralysis with rocuronium may theoretically aid in the prevention or mitigation of muscle fibre damage. However, at this point of time, no randomized controlled study has compared this matter with sufficient rigor.

Our study has some significant limitations. Firstly, we decided to utilize the MTT assay to draw conclusions about the number of viable muscle cells in our samples. As the assay measures the cytosolic oxidoreductase enzymatic activity, it allows a conclusion about the number of viable cells present. However, the assay does not allow any accurate prediction about the ability of cells to recover, hence it may or may not reflect the final count of permanent cell death. However, the decision to use the MTT assay was based on its previously described strong correlation with electron-microscopically detected morphologic signs of ischaemia-related muscle cell damage^22^. Secondly, all NMBA used in human anaesthesia do not only bind to nicotinic acetylcholine receptors, but also with much lower potency to muscarinic M_2_ and M_3_ receptors^23^. Though the low potency of rocuronium to act as an antagonist on M_2_ and M_3_ receptors^23^ makes it unlikely that clinically relevant effects may be observed, we cannot exclude that our results could have been influenced not only by the action of rocuronium on the muscle cell membrane, but also by extra-junctional receptor interaction. Furthermore has an influence of rocuronium on nitric oxide production been described^24^. The findings of this study by Baek *et al*.^24^ appear to correlate rocuronium with a pro-inflammatory pathway and offer no further explanation for our observations. However, our results are most likely explained by a reduction in cellular oxygen consumption due to neuromuscular paralysis. This study was designed to solely measure the effect of muscle relaxation on ischaemia-induced cell damage as a first proof of concept. We did not investigate the effect of post-ischaemia reperfusion. Lastly, our experimental design used isoflurane anaesthesia. Isoflurane, as all volatile anaesthetic agents is known to have mild to moderate inhibitory effects on both cardiac as well as skeletal muscles^25^. The clinically more significant effect of volatile anesthetics is however their interaction with NMBA. In general, they augment the effects of NMBA by about 20–30%^26^. As such, isoflurane could have influenced our results. However, as all rats were treated identically, and as all rats had an ischaemic as well as well as a non-ischaemic limb (hence acting as own control) it is unlikely that the presence of isoflurane was a significant trigger for our results.

## Conclusion

Neuromuscular paralysis with the NMBA rocuronium showed a strong protective effect in a setting of *in-vivo* induced muscle ischaemia in rats. As NMBA are commonly used during general anaesthesia, and as deep neuromuscular block prior to surgically induced ischaemia can easily be achieved, the results may have clinical implications. However, further experiments in humans are required to verify the clinical relevance.

## Electronic supplementary material

supplement 1
