# Supplementary material for: Effects of muscle relaxants on ischaemia damage in skeletal muscle
Source: Sci Rep. 2018 Apr 11;8:5794. doi: 10.1038/s41598-018-24127-2 (PMC5895809; doi:10.1038/s41598-018-24127-2)

**Effects of muscle relaxants on ischaemia damage in skeletal muscle**

Thomas Ledowski^1,*^, Simone Nißler^2,$^, Manuel Wenk^2,#^, Esther M. Pogatzki-Zahn^2,**^, Daniel Segelcke^2,##^

^1^ Anaesthesiology Unit, Medical School, The University of Western Australia, 35 Stirling Highway, Crawley WA 6009

^2^Department for Anaesthesiology, operative Intensive Care and Pain Medicine, University Hospital Muenster, Albert-Schweitzer-Campus 1, A1, 48149 Muenster

^$^ simone.nissler@web.de

^#^ Manuel.Wenk@ukmuenster.de

** pogatzki@anit.uni-muenster.de

^##^ Segelcke@anit.uni-muenster.de

*Corresponding Author: Prof. Thomas Ledowski, School of Medicine and Pharmacology, University of Western Australia, Level 2 Royal Perth Hospital MRF Building, Rear 50 Murray Street, Perth WA 6000, Australia

email: [Thomas.ledowski@health.wa.gov.au](mailto:Thomas.ledowski@health.wa.gov.au); phone: + 61 8 9224 0201

Institution: University Hospital Muenster, Albert-Schweitzer-Campus 1, A1, 48149 Muenster, Germany and The University of Western Australia, 35 Stirling Highway, Crawley WA 6009, Australia


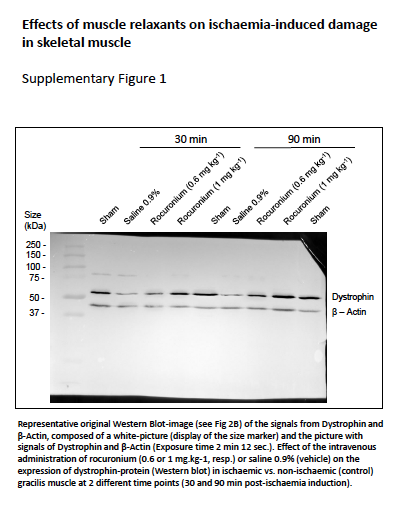

Supplement: Supplementary file 1 — supplement 1 [file 41598_2018_24127_MOESM1_ESM.docx]
